# Clicker Training Accelerates Learning of Complex Behaviors but Reduces Discriminative Abilities of Yucatan Miniature Pigs

**DOI:** 10.3390/ani10060959

**Published:** 2020-05-31

**Authors:** Pedro Paredes-Ramos, Joanna V. Diaz-Morales, Manuel Espinosa-Palencia, Genaro A. Coria-Avila, Apolo A. Carrasco-Garcia

**Affiliations:** 1 Facultad de Medicina Veterinaria y Zootecnia, Universidad Veracruzana. Miguel Ángel de Quevedo s/n esq. Yáñez, Col. Unidad Veracruzana, Veracruz 91710, Mexico; victoria_diazm4@hotmail.com (J.V.D.-M.); mvzmep@gmail.com (M.E.-P.); acarrasco@uv.mx (A.A.C.-G.); 2 Centro de Investigaciones Cerebrales, Universidad Veracruzana. Médicos 489, U.H. del Bosque, Xalapa-Enríquez 91010, Mexico; gcoria@uv.mx

**Keywords:** animal training, clicker training, pig cognition, object discrimination

## Abstract

**Simple Summary:**

Animal training is intended to teach specific behavioral responses to specific requests. Clicker Training (CT) is a method to train animals based on the use of a device that emits a sound to be associated as a marker that predicts the delivery of something wanted (food). It is believed that CT decreases training time compared to other types of training that use different markers, such as voice. Herein, we used two-month-old miniature piglets to assess whether CT decreased the number of repeats required to learn complex behaviors compared to voice-trained animals. Furthermore, we compared the number of correct choices of animals from both groups when tested for the discrimination of objects. The results indicated that CT decreased the number of repetitions required to learn to fetch an object but reduced the animals’ ability to make correct decisions during discriminatory trials compared to voice-trained animals. This suggests that CT is more efficient than voice in teaching complex behaviors but reduces the ability of animals to use the cognitive processes necessary to discriminate and select objects associated with reward. Animal trainers might consider our results to decide which marker is to be implemented based on the aim and purpose of the training.

**Abstract:**

Animal training is meant to teach specific behavioral responses to specific cues. Clicker training (CT) is a popular training method based on the use of a device that emits a sound of double-click to be associated as a first-order conditioned stimulus in contingency with positive reinforcements. After some repetitions, the clicker sound gains some incentive value and can be paired with the desired behavior. Animal trainers believed that CT can decrease training time compared to other types of training. Herein, we used two-month old miniature piglets to evaluate whether CT decreased the number of repetitions required to learn complex behaviors as compared with animals trained with voice instead of the clicker. In addition, we compared the number of correct choices of animals from both groups when exposed to object discriminative tests. Results indicated that CT decreased the number of repetitions required for pigs to learn to fetch an object but reduced the ability of animals to make correct choices during the discriminate trials. This suggests that CT is more efficient than voice to teach complex behaviors but reduces the ability of animals to use cognitive processes required to discriminate and select objects associated with reward.

## 1. Introduction

Animal training methodologies are commonly based on the principles of operant conditioning [1]. Operant conditioning is a type of learning whereby an association is formed between an arbitrary behavioral response and the immediate consequences. For example, a pigeon presses a lever and such response is followed by the delivery of food [2]. When a behavior is reinforced with a positive consequence, it will increase in frequency or will be more likely to occur in the future [3]. By contrast, when a behavior is not followed by a positive consequence, it will not be reinforced. Interestingly, delay in the delivery of primary reinforcers such as food can impair the rate at which animals learn to perform new tasks [4]. However, some evidence indicates that the learning impairment can be reduced if a signal (a secondary reinforcer) that predicts the primary reward is presented during the delay. For instance, in one study, rats provided with an immediate reward-predicting signal during the delay learned a discrimination task in about 20 trials, whereas control animals in a 5-s delayed reinforcement schedule required a median of 580 trials [5]. Accordingly, animal training can be improved by providing secondary reinforcers during the delay period.

Clicker training is one of the most popular animal training methods around the world [6,7]. The method was popularized by Pryor [8] using a small device that emits a sound of double-click that, if repeatedly paired with food, can bridge successive approximations to the wanted behavior. Overall, the clicker is pressed when a desired behavior occurs, and is typically followed by the presentation of food (as a primary reinforcer) as soon as possible [6,7,8,9]. After some repetitions, the clicker-sound can be associated with a reward and becomes a conditioned stimulus (CS) and a secondary reinforcer (SR) [2,10]. In applied settings, it has been proposed that clicker training can decrease the required time to perform a task and can also help animals be more motivated to work during a training trial [6,7,11,12]. Nevertheless, some studies in dogs, cats, and horses suggest that clicker training does not decrease training time compared to other SRs or a primary reinforcer alone [1,13,14,15,16]. On the other hand, one study showed that using a sound as SR in dwarf goats (*Capra hircus*) resulted in higher rates of task acquisition compared to animals trained with a primary reinforcer alone [17]. Thus, we believe that the mixed results in the capacity of an SR and clicker to improve learning in animals might be a consequence of the complexity of the trained behavior. For instance, learning of a simple behavior such as touching a target or pressing a lever can be taught by simply capturing the behavior, whereas complex behaviors cannot be simply captured and may require shaping. Despite these mixed results and the industry-reported benefits [7,11,12], it seems premature to conclude that a clicker does not facilitate animal training.

In applied settings, animal trainers tend to replace the clicker-sound by their voice as a verbal marker such as “yes” or “good dog” [7,12]. Nevertheless, according to Pryor [7], a clicker-sound is better than human voice as SR for several reasons: first, the human voice evolved to communicate many messages including emotions, and as a consequence, the same word uttered with different intonation may have different meanings [18]. Second, animals exposed to human interaction may create associations between the human voice and positive or aversive events and, as a consequence, one word would have different meanings and might not be a clear marker during training. As far as we know, only one unpublished master’s thesis [19], supervised by Karen Pryor (clicker developer), reported that clicker trained dogs achieved behavior in fewer minutes and required fewer repetitions than dogs trained with the word “yes” as an SR [19]. Altogether, it seems important to continue exploring the use of the clicker as a reinforcer. Herein, we used piglets to compare the amount of training required to shape a complex behavior with clicker or voice as an SR. In addition, we evaluated the ability of those animals to select correct choices in a discriminative test. Since learning to fetch an object requires both simple and complex behaviors, we predict that clicker training might decrease the number of repetitions required to achieved complex but not simple behaviors and impact the animals’ ability to make correct decisions during discriminatory trials.

## 2. Materials and Methods

### 2.1. Animals

We used 20 two-month-old Yucatan miniature piglets (*Sus scrofa domesticus*) of different sexes (eight males, 12 females). Animals were obtained from six different litters, weaned at 21 days of age. Weaned piglets were housed in groups of five in large corrals (4 m^2^) at the facilities of the Faculty of Veterinary Medicine, University of Veracruz, Mexico. Animals housed together belonged to the same experimental group. Piglets were fed once a day with corn-soybean diet formulated in the farm according to the Nutrient Requirements of Swine for growing pigs [20], and water available ad libitum. All individuals were habituated to human contact by authors J.V.D.-M. and M.E.-P. starting at postnatal day 6. Approval from the Faculty of Veterinary Medicine, University of Veracruz, Mexico, Animal Care and Use Committee for the use of animals was obtained prior to the start of the study. Before initiation of the study, all authors completed an education program for the care and use of research animals.

### 2.2. Groups

Animals were randomly divided in two groups. One group was trained and evaluated using a clicker as the SR, whereas the second group was trained with voice.

### 2.3. Apparatus

**Figure 1:**
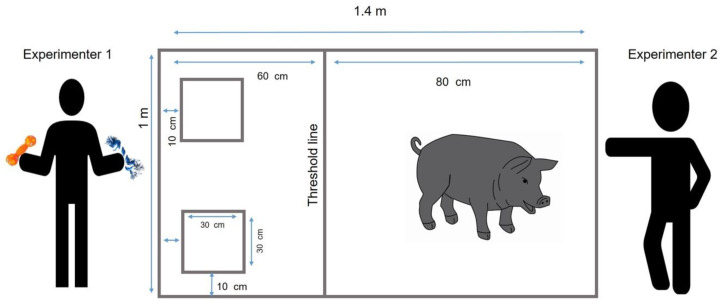
Object discrimination was evaluated into a rectangle drawn on the floor. Objects were placed into the 30 cm squares. Object selection occurred when piglets held an object and crossed the threshold line walking toward experimenter 2.

All training and experimental sessions occurred in a hall of 2 × 4 m, in which we used 3M duct tape (5 cm width) to draw a 1.4 m long × 1 m wide rectangle on the floor. Inside the rectangle, we drew two 30 cm squares with 10 cm margin on its corresponding side (left or right) and on top. Sixty centimeters from the same side, we placed a line that divided the rectangle and acted as a threshold line for the object selection (Figure 1).

### 2.4. Objects

We used objects that ranged in size from 10 to 15 cm^3^, including toys, kitchen instruments, puppets, dummies, and dolls made of different textures such as plastic, rubber, cloth, et cetera.

### 2.5. Training to Fetch (Steps and Criterion)

The purpose of training trials was to teach each piglet to fetch an object placed on the floor. Training sessions were limited to 30 repetitions per session, with one session per day. If piglets failed to achieve the criterion in one session, they started the next session at the same step. We used 1/4 (1–2 g) of a commercial biscuit (Marías™ Veracruz, Mexico) as a primary reinforcement. All training and testing were conducted by experimenter 1 (P.P.-R.). During each session, we used different objects in order to accustom animals to manipulate diverse textures, forms, weights, and sizes. We intended to expose the piglets to novel stimuli, to reduce the neophilic tendencies to explore a novel stimulus during discriminative tests. All correct responses by pigs in the clicker group received a clicker sound that was followed immediately by food placed in the floor. Likewise, all correct responses by animals in the voice group were marked with the word “very good” and followed by the primary reinforcer. In both conditions, the trainer delivered food 1 s after the SR.

Training occurred in 10 successive steps. The criterion was attained at each step before continuing onto the next step. Criterion one was achieved when piglets maintained attention to the trainer (to watch the hands or face of the trainer) for at least 5 s in five consecutive repetitions. In step two, the trainer introduced an object (e.g., toy, tube, fork, spoon, etc.) and the pig’s behavior was reinforced to look at or explore it. Criterion two was achieved when animals maintained attention to the object for at least three 5-s trials. Step three consisted of trials in which the trainer lured the pig to look at the object by holding food in front of it. When pigs touched the object, the trainer reinforced the behavior; criterion three was achieved when animals bit the object or put it into their mouth for at least five consecutive trials. During the next step, the trainer continued to reinforce object mouth manipulation; criterion four was achieved when pigs picked it up for at least 2 s during three consecutive trials. In step five, the trainer continued reinforcing the animal by holding the object; the criterion was attained when pigs kept the object in their mouth for at least 5 s in five consecutive times during the same session. In step number six, the trainer continued reinforcing the pigs by holding the object, and used luring to motivate the animal to turn its body toward the trainer while the object remained into its mouth. Criterion six was achieved when pigs picked up the object and turned their body a minimum of 90 degrees toward the trainer for three consecutive trials. During the next step, the trainer continued reinforcing the same behavior, but reinforced it only if the pig turned its body 180 degrees toward the trainer. Criterion seven was achieved when pigs responded with that behavior for at least five consecutive trials. Criterion eight was achieved when the pig turned its body while holding the object and walked two steps toward the trainer three consecutive times. During step nine, the trainer added the cue “fetch,” and the criterion was obtained when pigs picked up the object, turned, and walked, holding the object for at least 80 cm toward the trainer. Criterion 10 was achieved when pigs started to pay attention to the trainer and fetched the object only when the trainer pronounced the word “fetch.”

### 2.6. Discriminative Test

Once subjects learned to fetch and attained the criteria, they were exposed to 15 discrimination tests consisting of one novel reinforced object (CS+) and six discriminative trials. Each pig walked voluntarily to the testing area, which was 20 to 30 m away from its pen. At the beginning of every trial, piglets were placed inside the rectangle at the opposite side of the squares, facing the experimenter 2, who commanded “fetch” to ask the pigs to select one object (as shown in Figure 1). Experimenter 1 stood next to the testing area and was responsible for placing and replacing the objects. After every trial, experimenter 2 recovered the attention of the piglet, while experimenter 1 placed the objects again in the squares. While the piglets were choosing the object to be fetched, experimenter 1 turned his body to avoid communicate clues and to prevent a Clever Hans effect. First, one novel object was randomly placed on one square, and it was reinforced (CS+) when animals fetched it. Six subsequent discriminative tests consisted of placing the CS+ in one of the squares along with a novel object in the other square. The same CS+ object was used during all the discriminative trials of the same test, but it was not reused in the next tests. To prevent odor contamination, experimenter 2 retrieved the chosen object, and every time piglets fetched the CS+, it was replaced by a replica of the same object. Piglets were free to manipulate both objects before choosing one. We considered that subjects had chosen the object once they crossed the threshold line in front of trainer 2. In order to prevent spontaneous preferences for a single stimulus, the same kind of objects were used for animals from both groups but in a different order. Sometimes the object used as CS+ in an individual was the novel object in a different one. Before every session, the objects were randomly placed on the left or right.

### 2.7. Analyses

We used a factorial ANOVA to identify the main effects in the number of repetitions required to learn to fetch an object by group and criteria, as well as by their interaction. Likewise, factorial ANOVA was used to evaluate main effects in the number of correct choices by group, correct choices during trials of the same session, and their interaction. For specific comparisons between samples, the Tukey post hoc test was used. In all comparisons, a significance level of *p* < 0.05 was used. Analyses were performed using the Statistical Package statistic v11.

## 3. Results

**Figure 2:**
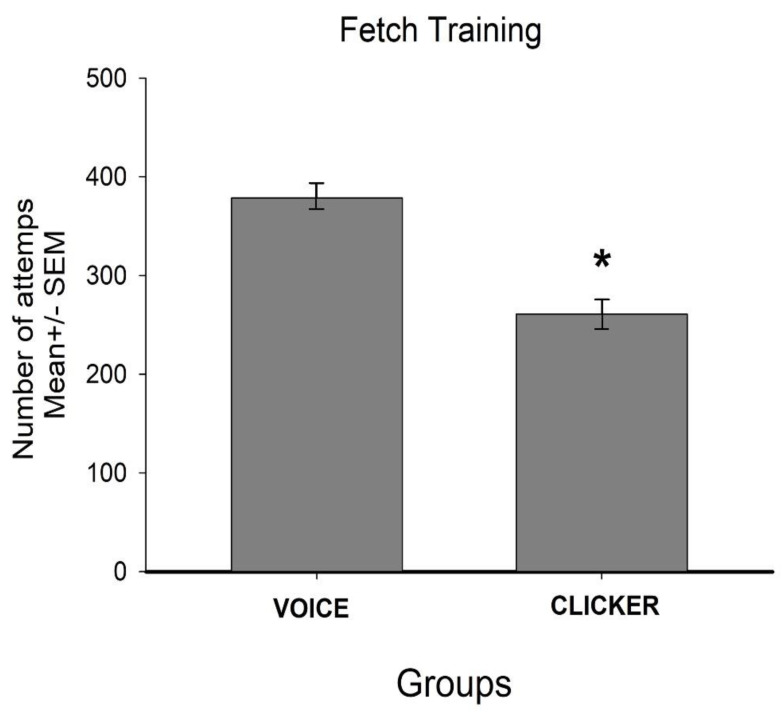
Animals from the clicker group required fewer repetitions to learn to fetch an object than those trained with voice. * indicates *p* < 0.05.

**Figure 3:**
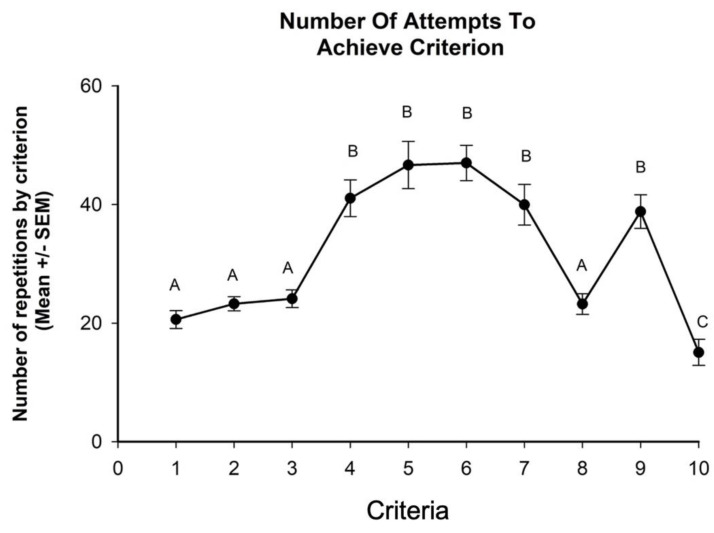
Fetch training was divided in 10 successive criteria. Piglets needed to achieve the criteria in order to progress to the next step. Statistical analysis detected differences in the number of repetitions required to achieve criteria. Different capital letters indicate significant differences *p* < 0.05.

**Figure 4:**
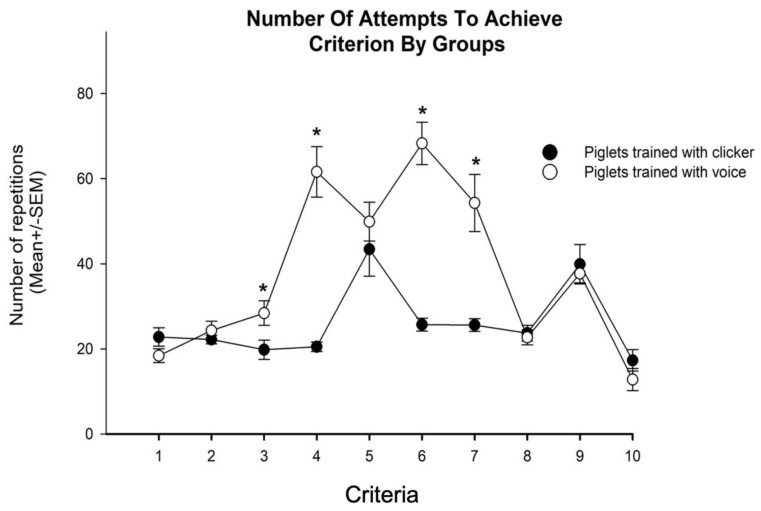
Piglets trained with voice required more repetitions than those trained with a clicker in criterion 3, 4, 6, and 7. No differences were observed in the rest of the criteria. This suggests that animals trained with a clicker required fewer number or repetitions to learn certain behaviors compared with animals trained with voice. * indicates *p* < 0.05.

With regard to the total number of repetitions required to learn to fetch an object, the ANOVA detected differences by group F (1.18) = 56.65, *p* < 0.05. Post hoc tests indicated that animals from the voice group required more repetitions than animals from the clicker group (Figure 2). In addition, the ANOVA identified differences between criteria F (9.18) = 23.06, *p* < 0.05, and in the number of attempts required to achieve criteria by groups F (9.180) = 14.195, *p* < 0.05 (Figure 3 and Figure 4).

**Figure 5:**
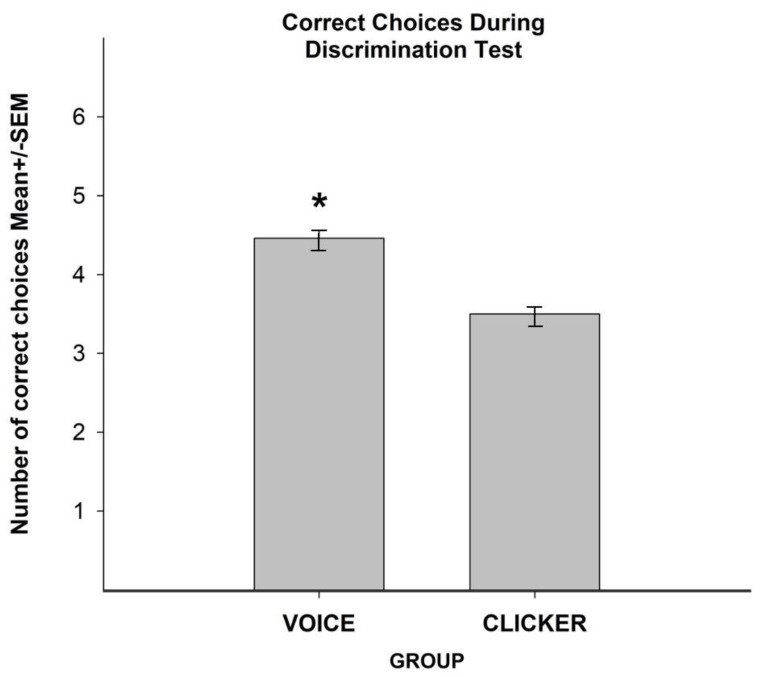
Piglets trained with voice displayed more correct choices during object discrimination trials compared with those trained with a clicker. * indicates *p* < 0.05.

With regard to the discrimination tests, the ANOVA identified differences by group F (1.18) = 54.0, *p* < 0.05. Post hoc tests showed that animals from the voice group displayed more correct choices than animals from the clicker group (Figure 5). No differences were observed by trials F (5.90) = 0.7946, *p* > 0.05 and by the interaction F (5.1908) = 0.20, *p* > 0.05.

## 4. Discussion

The present study was designed to compare the effectiveness of clicker training and voice as SRs, as well as to identify the effect of clicker training on discriminative abilities in pigs. The results indicated that piglets trained with a clicker-sound required fewer repetitions to learn to fetch an object compared with animals trained with voice. By contrast, the discriminative tests showed that piglets trained with voice displayed more correct choices than animals trained with clicker. Overall, these data suggest that clicker training facilitates the construction of complex behaviors by modeling sequential approximations but decreases the ability of animals to apply different strategies to solve problems in new complex tasks.

### 4.1. Clicker for Complex Behaviors

Generally, complex behaviors are built from a series of smaller components that trainers can adjust toward the definitive behavior [3]. Basic elements of modern animal training include capturing, shaping, and luring behaviors. While luring is used to guide animals towards the desired behavior and then reinforce it, capturing consists in reinforcing the behavior once it is observed. Given that some behaviors are infrequently or are never displayed by an individual, shaping can use luring and capturing to reinforce successive closer approximations to unlike behavior [6,10,21]. According to some studies [11,12,22], animal trainers consider that dogs trained with a clicker learn more quickly and that a clicker is easier to use to teach behaviors than training with a primary reinforcer alone. However, studies in controlled situations have struggled with finding those mentioned benefits [1,13,14,15,16]. To compare the efficiency of a clicker and voice, we trained piglets to fetch an object. In pigs, learning to fetch an object is so complex that it had to be divided in 10 progressive criteria. We considered that besides the complete sequence of fetching an object, other “small” behaviors used to build the fetch behavior in pigs might be considered as simple or complex depending on the difficulty to teach them. Accordingly, we considered as “simple” those criteria that could be achieved by simply capturing the behavior, and as “complex” the criteria that included behaviors that had to be lured and shaped. For instance, maintaining attention to the trainer (criterion 1) or to the object (criterion 2) were considered as simple behaviors because they were taught by capturing, whereas learning to pick up an object (criterion 3) and turning its body a minimum of 90 degrees toward the trainer while holding an object (criterion 6) were considered complex. Interestingly, the results indicated that while the number of repetitions required to achieve simple criteria (1, 2, 5, 8, 9, and 10) was similar in both groups, pigs from the voice group required a higher number of attempts to learn complex behaviors, as in criteria 3, 4, 6 and 7. Although behaviors in criteria 5 and 8 seem complex, they are in fact simple, because they were already built in the previous criteria (4 and 7), and during those steps, the trainer only captured longer duration. Likewise, we considered criteria 9 and 10 as simple because in both cases the fetch behavior was already built, and training consisted of adding the command or cue to trigger the behavior. On the other hand, if we consider the complete sequence of fetching an object as a single behavior, our results indicate that piglets from the clicker group required fewer repetitions to learn it compared to animals from the voice group. Because some studies [1,13,14,15,16] indicate that clicker training does not decrease training time used to achieve simple behaviors, such as nose touching a target [16] or lever pressing [1], we believe that the complexity in a given trained behavior emphasized the differences in effectiveness between the clicker and other forms of training. Altogether, it suggests that the use of a clicker can accelerate the learning of complex behavior, but does not have the same effect on the simple behavior. Even without knowing the precise mechanism by which the clicker may improve or facilitate learning in animals, we consider that the current evidence supports its use for training of complex chains of behavior.

### 4.2. Clicker Training Hypothesis

At least three mechanisms have been proposed to explain how clicker training works. They have been proposed as the reinforcing hypothesis (RH), marking hypothesis (MH), and bridging hypothesis (BH) [1,7,23]. The RH proposes that the SR clicker becomes a sort of “reward” itself and, as a consequence, is able to increase the likelihood of a behavior to occur [2]. With respect to the MH, it is argued that the clicker acts as a marking signal that helps animals distinguish what particular behavior is linked to the primary reinforcer [2,3]. Finally, the BH suggests that the clicker can fill the time between the behavior and the arrival of the primary reinforcer [24]. According to the characteristics of our study, we cannot discuss separately the mechanism by which the clicker outperformed voice. Nevertheless, we speculate that unlike a primary reinforcer (e.g., food) that has intrinsic value, an SR is at the beginning a neutral stimulus that acquires a particular incentive value after being repeatedly paired with a primary reinforcer [2]. Because piglets from our study were in contact with people from birth and were familiarized to human contact from postnatal day six, we believe voice was disadvantageous compared with clicker. Namely, verbal cues and human voice may have had meaning, and thus, its role as SR, marker, and bridge might not be as clear and efficient as the clicker-sound, considering that some studies indicate that pre-exposure to a particular stimulus can affect its salience and reduce its associability [25]. In addition, the correct responses in animals from the group voice were marked by saying the word “very good,” and because word pronunciation has a longer latency than clicker sound, we believe that the clicker had better timing than verbal markers to indicate to the subject what behavior was reinforced. Further investigation is required to evaluate the physical characteristics of the clicker and voice as SRs, markers, or bridges.

### 4.3. Discrimination

We used object discrimination (OD) to evaluate the effect of clicker training on decision making in piglets. Discrimination refers to the ability to differentiate stimuli on the basis of attributes of those stimuli through differential reinforcement contingencies [26]. Since OD allows animals to make classification and concepts, it is believed that it can act as a cognitive platform for other cognitive capacities. Animals are able to learn to discriminate objects, and these capacities diverge from simple to complex and even abstract concepts [17,27,28]. With regard to pigs, complex object and social discrimination abilities have been observed in situations that require the use of multiple memories [29,30]. For instance, pigs are able to remember the characteristics of particular objects two days or more after exposure and show preference for novel objects over familiar ones [30]. In addition, when pigs need to decide between one of two food sources, most of them prefer the one with more food and remember their location [31].

Most common discrimination tasks consist of a setting where an animal is presented with two stimuli, of which one is arbitrarily assigned as correct. Typically, the subject learns the correct response because only that response is reinforced. To succeed in our study, piglets were required to recognize the physical characteristics of the objects and memorize the CS+. The results indicated that animals trained with voice selected more consistently the CS+ than those trained with clicker. This suggests that animals trained using voice were better in discriminating the characteristics of the object and inferring which one was the correct option during the OD tests. Since animals trained with clicker tend to become more operant and proactive [7], we believe that during OD, piglets from the clicker group were more impulsive to repeat the fetch behavior to obtain the reward instead of spending time exploring and selecting the correct option. Nevertheless, more studies are required to clarify the effect of clicker training on the impulsivity of animals and the use of others cognitive processes.

On the other hand, during the OD test, piglets were presented with the CS+ object along with a novel object. To face situations where only part of the information about a problem is accessible, several studies [32,33,34,35,36,37] have shown that animals are able to use inference by exclusion (IE). IE refers to the ability to choose one option based on the systematic exclusion of alternatives [32]. In other words, assuming that the options are A or B, if it is not A, it must be B. Thus, a potential explanation for our results is that piglets use IE to simply avoid novel objects to find the correct option. However, if piglets use IE during a discriminative test, we would expect no main effect of the group, and the number of correct choices would increase during the trials and session. However, statistical analyses indicated that piglets trained with voice had more correct choices than those trained with the clicker, which suggests that IE was not the main strategy used by piglets to select the correct choice in the OD tests. Altogether, our results indicate that although clicker training can decrease the training time required to learn complex behaviors, it may reduce the ability of animals to solve problems by themselves when the context of the test is similar to that used during training.

### 4.4. Pig Cognition

Increase our knowledge on pig cognition can have important implications at different levels. For instance, from a scientific point of view, the pig brain is similar to the human brain in terms of both anatomy and biochemistry, and therefore, represents a high value as an animal model for biobehavioral research [38,39,40,41]. On the other hand, pigs are one of the most widely consumed meats in the world. Unfortunately, due to the high demand in their meat, most production systems keep animals in intensive and crowded indoor environments where they are unable to express natural behavior, which increases stress and reduces their welfare [40]. Therefore, we consider that increasing our knowledge about pig cognition may inspire further research on how this animal’s mind works and how we should treat and house them in animal production systems [42].

## 5. Conclusions

Clicker training decreased the amount of repetitions required for piglets to learn to fetch an object compared with those trained with voice. Interestingly, animals assisted with voice displayed more correct choices than those trained with clicker during discriminative tasks. Our results suggest that clicker training can facilitate learning of complex behaviors, thus making our animals more likely to learn complex behaviors quickly; however, it may also hinder their ability to use different strategies to solve situations where the information about a problem is incomplete. Training with a clicker had a main effect in both learning and discriminative tests; thus, we can discard a Clever Hans effect in our study. To improve our understanding on the effect of clicker training on time learning, as well as other cognitive processes, future studies should consider comparing the performance of animals with varying levels of cognitive complexity under a similar methodology.
